# Supplementary material for: Long-term therapeutic silencing of miR-33 increases circulating triglyceride levels and hepatic lipid accumulation in mice
Source: EMBO Mol Med. 2014 Jul 18;6(9):1133–41. doi: 10.15252/emmm.201404046 (PMC4197861; doi:10.15252/emmm.201404046)
Supplement: Supplementary file 1 — Supplementary Figures and Table [file emmm0006-1133-SD1.pdf]

Long-term therapeutic silencing of miR-33 increases circulating triglyceride levels and hepatic lipid accumulation in mice

Leigh Goedeke, Alessandro Salerno, Cristina M. Ramírez, Liang Guo, Ryan M. Allen, Xiaoke Yin, Sarah R. Langley, Christine Esau, Amarylis Wanschel, Edward A. Fisher, Yajaira Suárez, Angel Baldán, Manuel Mayr and Carlos Fernández-Hernando.

<u>TABLE OF CONTENTS</u>

1- Supplementary Figure legends
2- Figure S1
3- Figure S2
4-Table S1

SUPPLEMENTARY FIGURE LEGENDS

Figure S1. miR-33 does not target the *SREBP1* and *HMGCR* 3'UTR. A) Human *SREBP1 and HMGCR* 3'UTR sequences. Paired sequences indicate predicted miR-33 binding sites. **B**) miR-33 binding site conservation across different mammals**. C**) Luciferase reporter activity in COS7 cells transfected with control mimic (CM) or miR-33 mimic (miR-33) and the human 3'UTR of *SREBP1*, and *HMGCR*. Data are the mean ±SEM and representative of ≥2 experiments in triplicate.

Figure S2. Enrichment of functional annotation terms. The differentially expressed proteins were enriched (FDR<0.001) for gene ontology (GO) terms (red), Swiss-Prot and Protein Information Resource (Sp pir) keywords (blue), sequence features (UP SEQ) (green) and KEGG pathways (orange). The bars represent the percentage of the proteins in that category that were also identified as differentially expressed in the proteomics screen.

Table S1. List of differentially expressed proteins in the livers of miR-33 ASO treated mice compared to control ASO treated mice.

Supplemental Fig S1

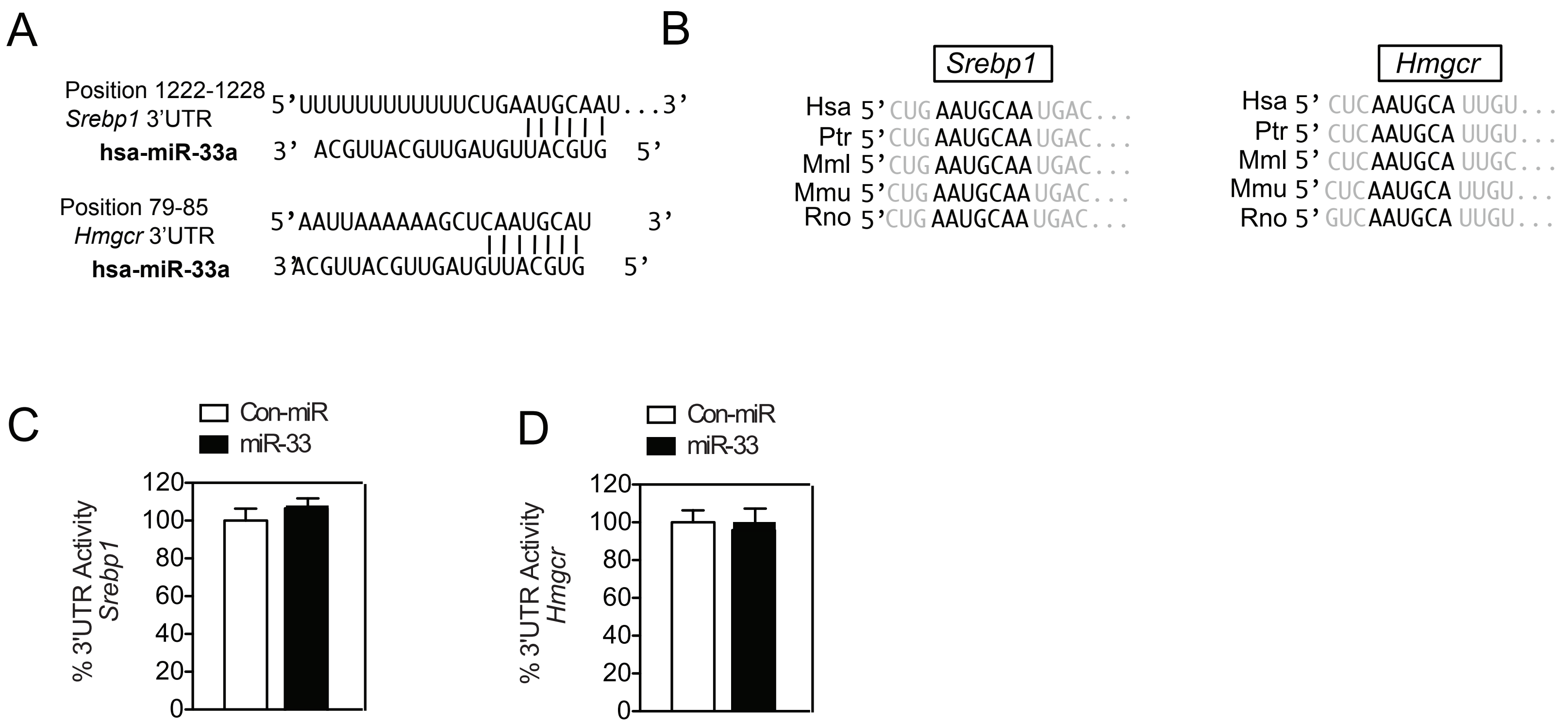

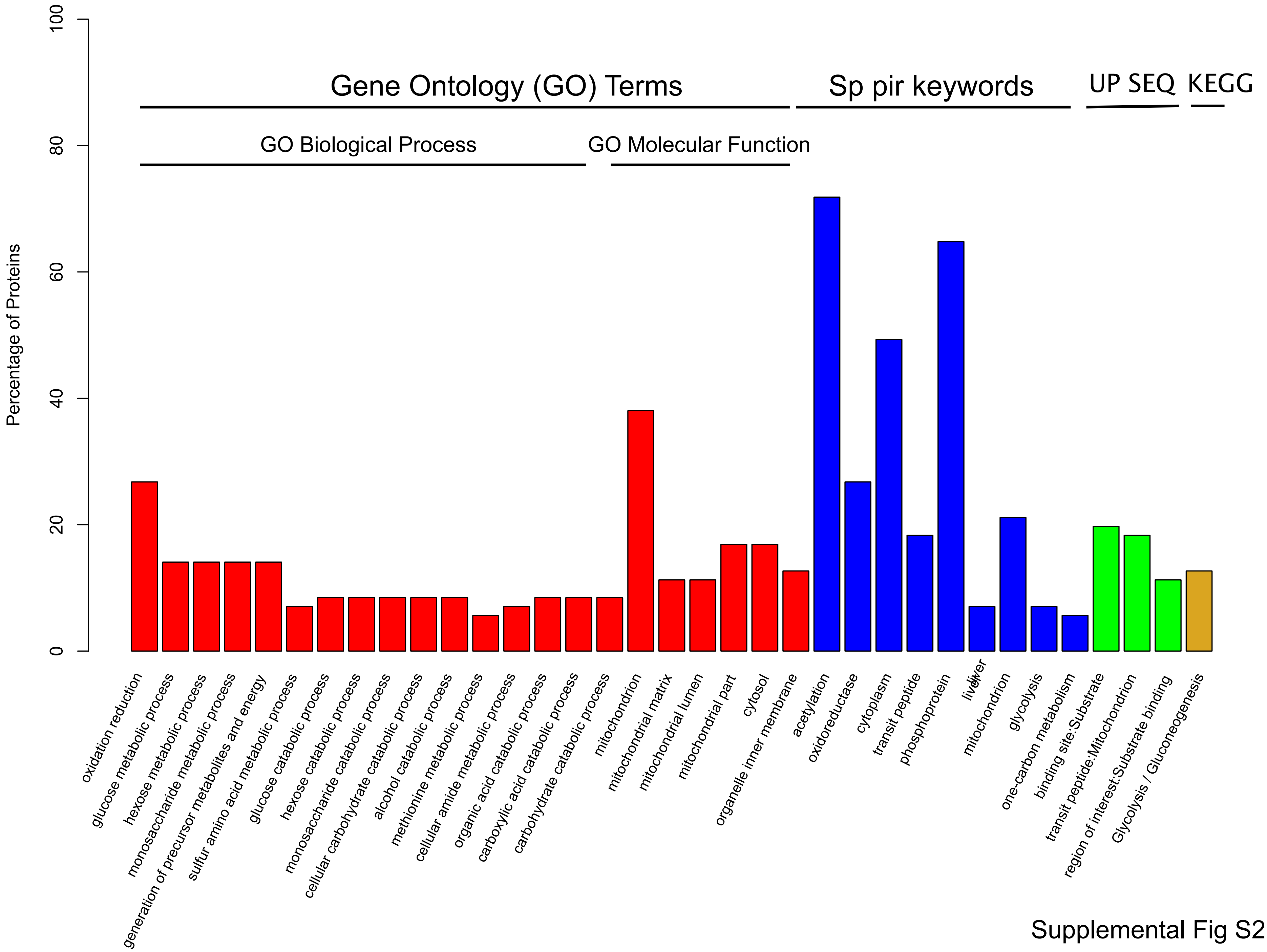

Supplemental Fig S2

No.	Protein name	Accession No.	Mw(Da)	T-test	Av. Ratio	No. of unique peptides	No. of unique spectra	No. of total spectra	Sequence coverage
1	Acyl-CoA-binding protein	ACBP_MOUSE	9,983	0.00069	-1.63	3	5	8	33.30%
2	Hemoglobin subunit beta-1	HBB1_MOUSE	15,822	0.02	-2.64	7	9	15	51.00%
3	Hemoglobin subunit beta-1	HBB1_MOUSE	15,822	0.041	-2.62	4	6	8	34.00%
3	Fatty acid-binding protein, liver	FABPL_MOUSE	14,228	0.041	-2.62	3	3	7	26.80%
4	Hemoglobin subunit beta-1	HBB1_MOUSE	15,822	0.036	-1.48	5	6	12	34.70%
5	Fatty acid-binding protein, liver	FABPL_MOUSE	14,228	0.0058	-3.01	4	5	9	33.90%
6	Glutathione S-transferase Mu 1	GSTM1_MOUSE	25,954	0.017	-1.34	5	6	12	21.10%
7	Cytochrome c oxidase subunit 5A, mitochondrial	COX5A_MOUSE	16,084	0.039	1.38	3	3	5	21.20%
8	Cytochrome c oxidase subunit 5B, mitochondrial	COX5B_MOUSE	13,795	0.0029	-1.28	5	6	11	29.70%
9	Fatty acid-binding protein, liver	FABPL_MOUSE	14,228	0.0076	1.34	4	4	8	27.60%
10	Hemoglobin subunit beta-1	HBB1_MOUSE	15,822	0.043	1.25	3	4	6	23.10%
11	Fatty acid-binding protein, liver	FABPL_MOUSE	14,228	0.036	-1.42	4	4	7	35.40%
12	Fatty acid-binding protein, liver	FABPL_MOUSE	14,228	0.014	1.27	8	11	27	52.80%
13	Single-stranded DNA-binding protein, mitochondrial	SSBP_MOUSE	17,300	0.011	-1.28	5	5	9	29.60%
14	Cytochrome b-c1 complex subunit 2, mitochondrial	QCR2_MOUSE	48,218	0.021	-1.42	2	2	4	6.62%
15	Eukaryotic translation initiation factor 5A-1	IF5A1_MOUSE	16,815	0.0015	1.34	8	10	19	35.70%
16	Myosin regulatory light chain 12B	ML12B_MOUSE	19,762	0.032	1.78	6	6	11	34.30%
17	Transcription factor BTF3 homolog 4	BT3L4_MOUSE	17,253	0.0025	1.52	4	6	8	22.80%
18	Major urinary protein 6	MUP6_MOUSE	20,632	0.001	5.14	8	12	20	42.80%
19	Major urinary protein 6	MUP6_MOUSE	20,632	0.013	1.92	9	12	17	50.00%
20	Major urinary protein 6	MUP6_MOUSE	20,632	0.011	2.88	6	8	14	33.30%
21	Major urinary protein 6	MUP6_MOUSE	20,632	0.01	2.93	13	17	30	76.10%
22	Major urinary protein 6	MUP6_MOUSE	20,632	0.0021	2.09	14	20	38	62.80%
23	Major urinary protein 6	MUP6_MOUSE	20,632	0.021	3.97	12	20	39	65.00%
24	Major urinary protein 6	MUP6_MOUSE	20,632	0.032	3.41	9	14	21	45.60%
25	Ferritin light chain 1	FRIL1_MOUSE	20,785	0.026	-1.8	8	12	32	59.00%
26	Peroxiredoxin-1	PRDX1_MOUSE	22,160	1.8E-07	-2.29	10	11	20	49.70%
27	Peroxiredoxin-1	PRDX1_MOUSE	22,160	0.000065	-1.66	13	13	23	61.30%
28	Carbamoyl-phosphate synthase [ammonia], mitochondrial	CPSM_MOUSE	164,603	0.00065	-1.45	6	7	12	5.13%
29	Peroxiredoxin-1	PRDX1_MOUSE	22,160	0.00037	1.33	13	14	21	66.80%
30	Protein DJ-1	PARK7_MOUSE	20,003	0.0021	1.2	8	12	21	42.30%
31	Apolipoprotein A-I	APOA1_MOUSE	30,569	0.0097	1.71	13	17	28	36.40%
32	Fructose-bisphosphate aldolase B	ALDOB_MOUSE	39,489	0.026	-1.33	7	8	16	23.10%
32	Peroxiredoxin-6	PRDX6_MOUSE	24,854	0.026	-1.33	6	7	11	32.60%
33	Fructose-bisphosphate aldolase B	ALDOB_MOUSE	39,489	0.0096	-1.29	8	10	16	23.90%
34	Carbonic anhydrase 3	CAH3_MOUSE	29,349	0.00059	-1.56	7	9	13	29.20%
34	Proteasome subunit alpha type-6	PSA6_MOUSE	27,355	0.00059	-1.56	6	8	11	26.40%
35	Electron transfer flavoprotein subunit beta	ETFB_MOUSE	27,605	0.011	1.21	10	12	22	44.30%
35	Enoyl-CoA hydratase, mitochondrial	ECHM_MOUSE	31,457	0.011	1.21	9	11	20	30.70%
35	3,2-trans-enoyl-CoA isomerase, mitochondrial	D3D2_MOUSE	32,061	0.011	1.21	7	9	16	26.30%
36	Ornithine carbamoyltransferase, mitochondrial	OTC_MOUSE	39,748	0.0049	-1.43	8	10	19	25.10%
37	Cathepsin D	CATD_MOUSE	44,937	0.016	1.41	3	3	7	9.27%
37	4-hydroxyphenylpyruvate dioxygenase	HPPD_MOUSE	45,037	0.016	1.41	4	4	7	11.20%
38	Glyceraldehyde-3-phosphate dehydrogenase	G3P_MOUSE	35,792	0.027	-1.91	6	8	15	19.50%
39	Fructose-bisphosphate aldolase B	ALDOB_MOUSE	39,489	0.0081	-1.24	11	12	26	28.60%
40	3-hydroxyanthranilate 3,4-dioxygenase	3HAO_MOUSE	32,786	0.042	1.38	5	6	8	19.60%
40	Copper chaperone for superoxide dismutase	CCS_MOUSE	28,893	0.042	1.38	5	5	7	21.20%
41	Pyruvate dehydrogenase E1 component subunit beta, mitochondrial	ODPB_MOUSE	38,919	0.034	-1.37	7	7	15	18.90%
41	Regucalcin	RGN_MOUSE	33,389	0.034	-1.37	7	7	13	27.40%
42	4-hydroxyphenylpyruvate dioxygenase	HPPD_MOUSE	45,037	0.018	-1.26	10	11	19	28.80%
42	Nicotinate-nucleotide pyrophosphorylase [carboxylating]	NADC_MOUSE	31,512	0.018	-1.26	7	9	14	27.40%
43	Ester hydrolase C11orf54 homolog	CK054_MOUSE	34,978	0.0056	-1.52	10	12	22	32.40%
44	Nicotinate-nucleotide pyrophosphorylase [carboxylating]	NADC_MOUSE	31,512	0.018	1.2	10	14	30	44.80%
45	L-lactate dehydrogenase A chain	LDHA_MOUSE	36,481	0.0049	-1.61	13	15	23	31.90%
46	Betaine--homocysteine S-methyltransferase 1	BHMT1_MOUSE	45,002	0.00042	-2.24	9	10	15	29.50%
46	Glycine N-methyltransferase	GNMT_MOUSE	32,658	0.00042	-2.24	7	9	14	33.80%
47	Uricase	URIC_MOUSE	35,022	0.0024	-1.68	6	10	18	24.80%
48	Heterogeneous nuclear ribonucleoproteins A2/B1	ROA2_MOUSE	37,385	0.0055	1.5	9	9	13	23.50%
49	Glyceraldehyde-3-phosphate dehydrogenase	G3P_MOUSE	35,792	0.0068	1.21	11	13	29	37.20%
50	Glyceraldehyde-3-phosphate dehydrogenase	G3P_MOUSE	35,792	0.0042	1.25	9	13	35	34.80%
51	Glyceraldehyde-3-phosphate dehydrogenase	G3P_MOUSE	35,792	0.0061	1.32	8	13	21	30.00%
52	Glyceraldehyde-3-phosphate dehydrogenase	G3P_MOUSE	35,792	0.015	1.33	8	12	19	27.30%
52	Estradiol 17 beta-dehydrogenase 5	DHB5_MOUSE	37,031	0.015	1.33	9	9	17	25.70%
52	Thiosulfate sulfurtransferase	THTR_MOUSE	33,448	0.015	1.33	7	10	17	29.30%
53	Glyoxylate reductase/hydroxypyruvate reductase	GRHPR_MOUSE	35,312	0.017	1.4	8	9	14	24.10%
54	Ornithine carbamoyltransferase, mitochondrial	OTC_MOUSE	39,748	0.01	1.29	9	10	23	28.00%
55	Transaldolase	TALDO_MOUSE	37,371	0.03	-1.23	11	13	29	32.90%
55	Fructose-1,6-bisphosphatase 1	F16P1_MOUSE	36,896	0.03	-1.23	11	15	27	33.40%
56	Fructose-bisphosphate aldolase B	ALDOB_MOUSE	39,489	0.016	-1.81	18	27	54	48.90%
57	Fructose-bisphosphate aldolase B	ALDOB_MOUSE	39,489	0.0044	1.43	15	22	46	46.40%
58	Fructose-bisphosphate aldolase B	ALDOB_MOUSE	39,489	0.015	1.47	15	19	36	44.80%
59	Fructose-bisphosphate aldolase B	ALDOB_MOUSE	39,489	0.039	1.24	10	14	24	31.90%
60	Sorbitol dehydrogenase	DHSO_MOUSE	38,231	0.02	-1.22	9	13	29	30.80%
60	Arginase-1	ARGI1_MOUSE	34,791	0.02	-1.22	10	13	24	40.90%
61	Leukocyte elastase inhibitor A	ILEUA_MOUSE	42,559	0.0094	-1.39	10	12	18	25.30%
62	Actin, cytoplasmic 1	ACTB_MOUSE	41,720	0.038	1.26	8	9	14	21.30%
63	Succinyl-CoA ligase [GDP-forming] subunit beta, mitochondrial	SUCB2_MOUSE	46,823	0.047	1.24	9	11	17	24.70%
64	Actin, cytoplasmic 1	ACTB_MOUSE	41,720	0.048	-1.67	9	10	17	25.10%
65	Keratin, type II cytoskeletal 8	K2C8_MOUSE	54,549	0.0091	-1.44	16	24	41	33.90%
66	Keratin, type I cytoskeletal 18	K1C18_MOUSE	47,521	0.00016	-1.87	13	16	28	24.80%
67	Keratin, type I cytoskeletal 18	K1C18_MOUSE	47,521	0.005	-1.55	15	21	34	34.30%
68	Keratin, type II cytoskeletal 8	K2C8_MOUSE	54,549	0.00017	-2.22	19	26	37	36.50%
69	Keratin, type I cytoskeletal 18	K1C18_MOUSE	47,521	0.037	-1.23	16	20	30	34.80%
70	Keratin, type I cytoskeletal 18	K1C18_MOUSE	47,521	0.00021	-1.44	15	22	38	34.80%
71	Keratin, type I cytoskeletal 18	K1C18_MOUSE	47,521	0.0043	1.63	24	35	61	55.60%
72	Alpha-enolase	ENOA_MOUSE	47,124	0.042	-1.21	4	4	7	8.76%
73	S-adenosylmethionine synthetase isoform type-1	METK1_MOUSE	43,490	0.003	-1.36	12	18	37	33.80%
74	Adenosylhomocysteinase	SAHH_MOUSE	47,671	0.046	1.29	12	16	28	25.70%
75	4-trimethylaminobutyraldehyde dehydrogenase	AL9A1_MOUSE	53,497	0.034	1.25	11	15	26	20.90%
75	Serine hydroxymethyltransferase, cytosolic	GLYC_MOUSE	52,568	0.034	1.25	9	10	18	19.70%
76	Homogentisate 1,2-dioxygenase	HGD_MOUSE	49,973	0.000045	1.35	8	10	18	19.30%
76	Hydroxymethylglutaryl-CoA synthase, mitochondrial	HMCS2_MOUSE	56,806	0.000045	1.35	8	10	16	14.20%
77	Trifunctional enzyme subunit beta, mitochondrial	ECHB_MOUSE	51,370	0.011	1.55	11	13	21	25.10%
77	Elongation factor 1-alpha 1	EF1A1_MOUSE	50,096	0.011	1.55	9	10	17	18.40%
78	Keratin, type II cytoskeletal 8	K2C8_MOUSE	54,549	0.0012	1.84	19	25	41	39.00%
79	Selenium-binding protein 2	SBP2_MOUSE	52,593	0.0049	1.37	23	29	51	53.40%
80	Selenium-binding protein 2	SBP2_MOUSE	52,593	0.022	1.37	25	33	60	55.70%
81	T-complex protein 1 subunit alpha B	TCPA2_MOUSE	60,432	0.023	1.34	22	27	47	40.50%
82	Phosphoglucomutase-1	PGM1_MOUSE	61,501	0.00023	1.23	12	13	23	26.20%
82	Carbamoyl-phosphate synthase [ammonia], mitochondrial	CPSM_MOUSE	164,603	0.00023	1.23	12	13	21	8.07%
83	Stress-induced-phosphoprotein 1	STIP1_MOUSE	62,567	0.0091	1.24	13	18	22	23.80%
84	Carbamoyl-phosphate synthase [ammonia], mitochondrial	CPSM_MOUSE	164,603	0.0013	1.23	18	20	34	12.50%
85	Stress-induced-phosphoprotein 1	STIP1_MOUSE	62,567	0.0061	1.26	12	15	21	26.50%
85	Lamin-A/C	LMNA_MOUSE	74,221	0.0061	1.26	14	15	20	17.90%
86	Bifunctional purine biosynthesis protein PURH	PUR9_MOUSE	64,200	0.022	1.28	11	14	20	23.00%
86	Electron transfer flavoprotein-ubiquinone oxidoreductase, mitochondrial	ETFD_MOUSE	68,074	0.022	1.28	9	9	15	17.40%
87	T-complex protein 1 subunit zeta	TCPZ_MOUSE	57,988	0.013	1.21	9	10	18	17.50%
88	Heat shock cognate 71 kDa protein	HSP7C_MOUSE	70,855	0.014	-1.28	7	7	8	14.90%
89	Sarcosine dehydrogenase, mitochondrial	SARDH_MOUSE	101,664	0.011	-1.21	8	9	14	11.10%
90	C-1-tetrahydrofolate synthase, cytoplasmic	C1TC_MOUSE	101,240	0.02	1.29	34	44	68	39.40%

Ratio is given for protein spot abundance in miR-33 ASO compared to control ASO
