# Supplementary figures and images for: Long-term therapeutic silencing of miR-33 increases circulating triglyceride levels and hepatic lipid accumulation in mice
Source: EMBO Mol Med. 2014 Jul 18;6(9):1133–41. doi: 10.15252/emmm.201404046 (PMC4197861; doi:10.15252/emmm.201404046)

Figure 1K

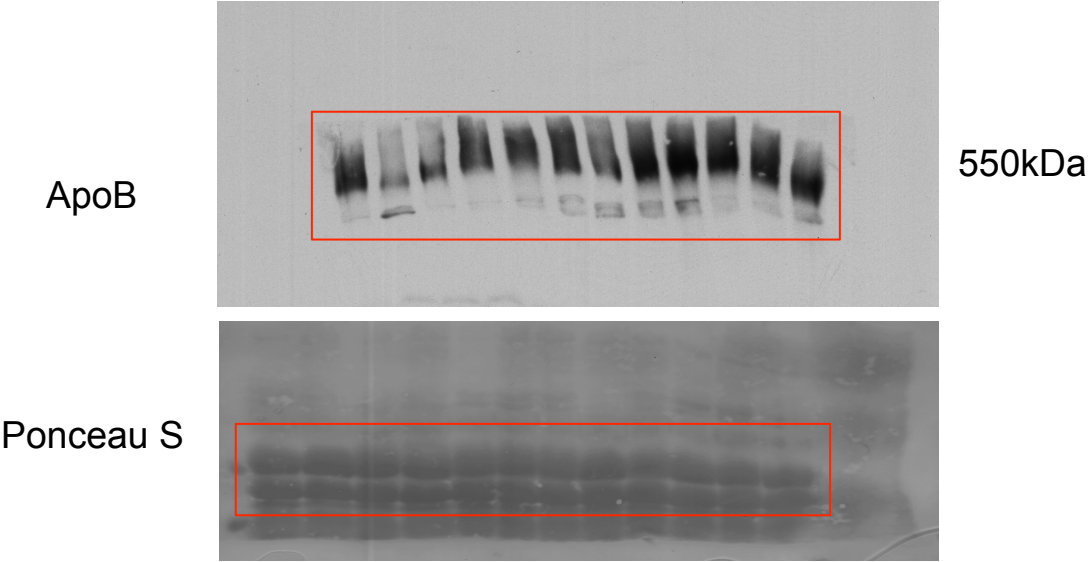

Supplement: Supplementary file 3 — Source Data for Figure 1K [file emmm0006-1133-SD3.pdf]

Figure 2K

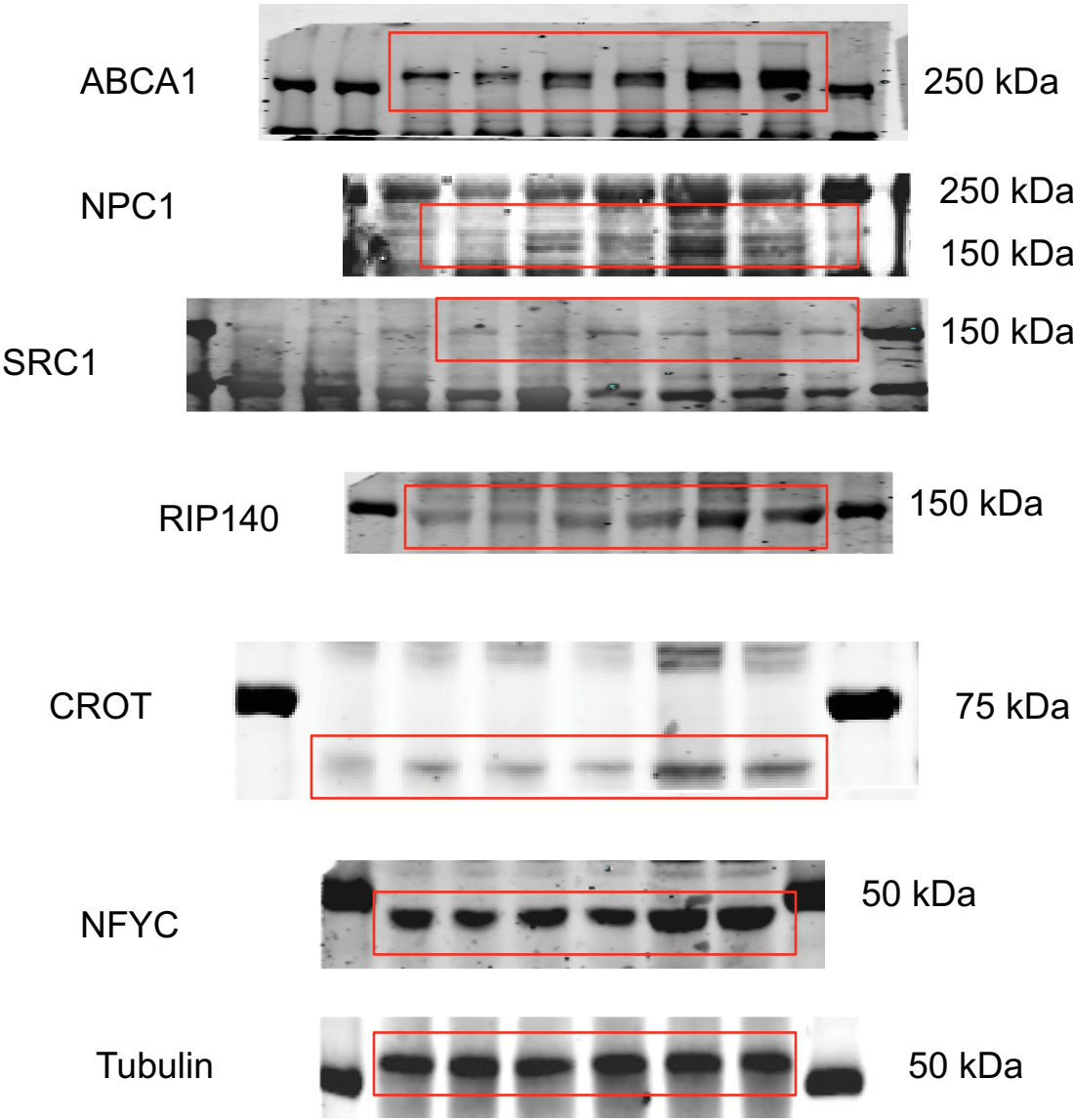

Supplement: Supplementary file 4 — Source Data for Figure 2K [file emmm0006-1133-SD4.pdf]
